# Supplementary material for: Reversal of splicing infidelity is a pre-activation step in B cell differentiation
Source: Front Immunol. 2022 Dec 19;13:1060114. doi: 10.3389/fimmu.2022.1060114 (PMC9806119; doi:10.3389/fimmu.2022.1060114)
Supplement: Supplementary Data Sheet 2 — PCR primers used in this study. [file DataSheet_2.pdf]

Gene	Forward Primer	Reverse primer
BAZ2A	GCCAGAAATAAGCGGAAACAAGA	ACTGGGCAATGTCAGACCAG
DDX47	AAGCTTGTGACCAGTTGGGA	TCGGCTTCATCCATGACCAA
MYCBP2	AGCCAGAGAGCAATATGAAGTCT	CTGGTGCAGGAGTCAGTGATG
SECISPBP2	AGTCCTGACAGTTCAAGAGCC	AGTCCAACTGCACTGGAAGC
SIPA1	GTGCTGGCTACTACCGCAAA	TCCAGAAGTTTCCTTGGGGAC
SMAD3	AGCTGTGTGAGTTCGCCTTC	CTGTGGTTCATCTGGTGGTCA
USP38	TGGCAGCTGTCCAGAAGTTT	GCTGTACTTGAAGGCAGACCA
